# Signalling in auctions for risk-averse bidders

**DOI:** 10.1371/journal.pone.0275709

**Published:** 2022-10-14

**Authors:** Olivier Bos, Francisco Gomez-Martinez, Sander Onderstal

**Affiliations:** 1 Université Paris-Saclay, ENS Paris-Saclay, CEPS and ZEW-Leibniz Centre for European Economic Research, Gif-sur-Yvette, France; 2 Department of Economics, University of Fribourg, Fribourg, Switzerland; 3 University of Amsterdam and Tinbergen Institute, Amsterdam, Netherlands; Universität Heidelberg, GERMANY

## Abstract

We study risk-averse equilibrium bidding in first-price and second-price sealed-bid auctions where bidders have signalling concerns, i.e., they care about how the auction outcome is interpreted by an outside observer. We find that when the winner’s identity and her payment are revealed to the outside observer, risk aversion yields less aggressive bidding behaviour in the second-price sealed-bid auction than in the risk-neutral case. Our analysis explains various revenue ranking reversals relative to the risk-neutral equilibrium observed in a recent experiment by Bos (2021).

## 1. Introduction

In the past two decades, auctions in which bidders care about how an outside observer interprets the auction outcome have been studied extensively. Applications include art selling, charitable fundraising, takeover bidding, competing firms issuing equity, and procurement. In such settings, bidders are expected to condition their bidding strategies on the signalling opportunities. Various disclosure policies, including revealing the winner’s identity, the (winning) bids, and the (winner) payments, have been shown to affect equilibrium bidding in auctions [1–6]. examine how revealing the winner’s identity and the bids to an outside observer affects equilibrium bidding [7–9] consider the effect of revealing the winner’s identity and the payments.

The received literature has mainly focused on the risk-neutral case, ignoring risk aversion. This is surprising as risk aversion has been shown to explain various observations in auctions better than risk neutrality, both in the lab and in the field [10–12] argue that risk aversion explains bidding behaviour in the laboratory. For field evidence, see [13–16].

A prominent example is the experimental observation in the symmetric-independent-private-values paradigm that the first-price sealed-bid auction (FPSB) yields higher revenue on average than the second-price sealed-bid auction (SPSB), violating the famous revenue-equivalence result for the case of risk-neutral bidders (see [17,18]). The recent experiment by [7], hereafter BGOT, highlights the need to understand the effect of risk-aversion in auction settings with signalling opportunities. In such a setting, BGOT observe that submitted bids deviate notably from the risk-neutral Bayesian perfect Nash equilibrium. More specifically, (i) bidders overbid/underbid systematically in FPSB/SPSB (in an experiment studying various disclosure policies in FPSB [6], also observe consistent overbidding relative to the risk-neutral equilibrium); (ii) FPSB yields higher revenue than SPSB; (iii) revenue in SPSB is higher when only the winner is revealed than when the winner and her payment are revealed.

In the current paper, we study theoretically whether risk-averse bidding can explain the anomalies observed in BGOT’s experiment. We obtain the following results. In FPSB, risk aversion leads to more aggressive equilibrium bidding compared to the risk-neutral case. Intuitively, like in the standard case where bidders’ utilities are not affected by how an outside observer interprets their bids, risk-averse bidders mitigate the risk of losing by submitting a higher bid than risk-neutral bidders. Moreover, when both the winner identity and her payment are revealed, risk-averse bidders have an additional incentive to bid aggressively because the winner gets a certain payoff from signalling opportunities while a loser’s payoff is random as the payoffs only depend on the winner’s bid. In SPSB, when only the winner’s identity is revealed, risk aversion does not affect the equilibrium in weakly dominant strategies. When both the winner identity and her payment are revealed, risk-averse bidders bid less aggressively than risk-neutral bidders. The reason is that winning in the SPSB is relatively unattractive because payoffs from signalling opportunities are random in that they depend on the second highest bid. The observation that risk aversion depresses bids in SPSB is in contrast to the standard independent-private-values model without signalling opportunities, where risk attitude does not affect the weakly dominant strategy of bidding value (see [19,20] for an analysis of equilibrium bidding for risk-averse bidders in the independent-private-values model). Our findings offer a potential explanation for the above experimental results in BGOT.

The remainder of this paper is organized as follows. In Section 2, we describe our model. Section 3 contains our findings. Section 4 is a short conclusion.

## 2. The model

We consider a setting with *n*≥2 (female) bidders, indexed *i* = 1,…,*n*, who compete for a single object allocated through an auction. Each bidder *i* is privately informed about her value *v*_*i*_ (her ‘type’). The *v*_*i*_’s are i.i.d. drawn from a distribution function *F* on $\left[0,\bar{v}\right]$, $\bar{v}>0$, which admits a continuous density function *f*≡*F*′ The auction outcome is partly revealed to an outside observer (male). We study settings where the outside observer either observes the winner’s identity and her payment, or only the winner’s identity. Our model reflects economic situations in which bidders care about how they are perceived by others. For instance, the fact that a firm wins or loses an auction could be interpreted by market analysts as a signal of the firm’s management quality. Another example is when outsiders consider a high bid in a charity auction as informative about how much the bidder cares about the charity. Our assumption that in some settings the winner’s payment is communicated to outsiders is not without application either: A directive on public procurement in the European Union stipulates, that payments to the bidders should be publicly revealed in a contract award notice (see Annex V part D of Directive 2014/24/EU of the European Parliament and of the Council of 26 February 2014 on public procurement).

We assume that bidders not only care about winning the auction, but also about the inference of the outside observer about her type. Following BGOT, we assume that a bidder’s payoff is increased by $\gamma \tilde{v}(\gamma >0)$, where $\tilde{v}$ denotes the outside observer’s best estimate of the bidder’s value based on what he learns about the auction outcome that is revealed. The resulting payoff for bidder *i* is given by

$$\pi_{i}=\left\{ \begin{array}{cc} v_{i}-p+\gamma \tilde{v} & if\;w=i\\ \gamma \tilde{v} & if\;w\neq i \end{array},\right.$$

where *w* denotes the auction winner and *p* the winner’s payment. The parameters in BGOT’s experiment are *γ* = 1/2, *n* = 3, and *F* = *U*[0, 1]. BGOT compare FPSB and SPSB in two settings. In the first setting, the winner’s identity is disclosed to the outside observer. This setting is labelled FPW and SPW for FPSB and SPSB respectively. In the other setting, labelled FPWP and SPWP respectively, both the winner identity and her payment are revealed to the outside observer.

We assume that bidders are expected-utility maximisers. All bidders evaluate their payoffs according to utility function *u*: ℝ→ℝ. In the remainder of the paper, we compare risk-neutral bidders, where *u*(*x*) = *x* for all *x*∈ℝ, to risk-averse bidders. As all bidders share the same beliefs about the other bidders’ values, we assume them to follow a symmetric bidding strategy. *B* denotes the strictly increasing risk-neutral bidding function and *A* denotes the strictly increasing risk-averse bidding function, with $B,A:[0,\bar{v}]\to R_{+}$ (to avoid messy notation, we do not use labels for auction, the case, or the level of risk aversion, unless indicated otherwise).

The risk-neutral analysis follows straightforwardly from [4,9]. While for risk-neutral bidders, the uniform distribution assures a unique fully separating equilibrium, it is unclear for risk-averse bidders. Therefore, we restrict our attention to the perfect Bayesian Nash equilibria that survive [21]’s D1 criterion (referred to as “equilibrium” in the remainder of this paper). Table 1 presents the risk-neutral equilibrium bids and expected revenue for the experimental parameters as well as the average revenue observed in BGOT’s experiment. Three observations stand out from the experimental findings: (i) overbidding/underbidding in FPSB/SPSB relative to the risk-neutral equilibrium predictions; (ii) comparing FPW to SPW, and FPWP to SPWP, FPSB yields higher revenue than SPSB, in contrast to the risk-neutral equilibrium predictions; (iii) revenue in SPSB is higher in SPW than in SPWP, in contrast to the risk-neutral equilibrium predictions. We investigate risk-averse bidding in an attempt to understand these three anomalies.

**Table 1:** Equilibrium bids and revenue for risk-neutral bidders for *γ* = 1/2, *n* = 3, *F* = *U*[0,1].

Case	Auction	Information to the outside observer	Equilibrium bids	Expected revenue	Average revenue in BGOT’s experiment
FPW	FPSB	The winner	$B(v)\approx \frac{2}{3}v+0.22$	*R*≈0.72	0.7319
FPWP	FPSB	The winner and her payment	*B*(*v*) = *v*	*R* = 0.75	0.8039
SPW	SPSB	The winner	*B*(*v*)≈*v*+0.22	*R*≈0.72	0.6830
SPWP	SPSB	The winner and her payment	$B(v)=\frac{v}{2}+5/8$	*R* = 0.875	0.6475

### 3. Risk-averse bidders

In this section, we explore risk aversion as a potential driver for more aggressive bidding in FPSB with signalling incentives and underbidding behaviour compared to predictions for SPSB and the consequences for the revenue ranking reversals. In order to do so, we derive the equilibrium bidding strategies for risk-averse bidders and compare them with the equilibrium bidding strategies for risk-neutral bidders (see [4,9]). In the case of risk-averse preferences, we assume that *u* is twice differentiable with *u*′>0 and *u*′′<0. Following [22], to simplify the analysis of SPSB when both winner’s identity and her payment are disclosed, we consider constant absolute risk aversion (CARA) preferences, i.e., *u*(*x*) = −exp(−*αx*)/*α* with *α*>0.

The remainder of this section is structured as follows. In Section 3.1, we establish the role of risk aversion in FPW and SPW. In Section 3.2, we establish that, relative to the risk-neutral case, risk aversion shifts equilibrium bidding functions upwards in FPWP. In Section 3.3, we derive equilibrium bidding functions with risk aversion in SPWP and show that risk aversion depresses equilibrium bidding under CARA preferences. Finally, in Section 3.4, we compare expected revenues in FPWP, SWP and SPWP assuming CARA preferences and show that for sufficiently risk-averse bidders, SPWP yields lower expected revenue than FPWP and SWP.

### 3.1 FPW and SPW

For the setting where only the identity of the winning bidder is revealed, let *k*_*w*_ and *k*_*l*_ denote the outside observer’s equilibrium value estimates for the winner and the losing bidders respectively. It is readily verified that *k*_*w*_ = 2^−1/*n*^ and that *k*_*l*_ is implicitly and uniquely defined by $\frac{k_{l}}{n-1}(n-{k_{l}}^{n-1})=\frac{1}{2}$, *k*_*l*_∈(0,1) (BGOT, Appendix A.4). Because these quantities do not depend on the bids submitted on the equilibrium path, the effect of risk aversion on equilibrium bidding follows from standard reasoning. In particular, in SPSB, bidding value plus *γ*(*k*_*w*_−*k*_*l*_) is the unique weakly dominant strategy, regardless of risk attitude. In FPSB, the (unique) risk-averse equilibrium is readily derived from the equilibrium in the standard setting without outside observer by inflating all values by *γ*(*k*_*w*_−*k*_*l*_). As a result, the standard result that FPSB generates a higher revenue than SPSB in the case of risk-averse bidders generalizes to our setting if only the winner’s identity is revealed. BGOT’s experimental observations are in line with this result (see Table 1), although the difference between the auctions is not statistically significant.

#### Proposition 1

*Suppose only the winner’s identity is revealed to the outside observer. Then, (i) in FPSB, risk-averse bidders bid more aggressively in equilibrium than risk-neutral bidders, (ii) equilibrium bidding in SPSB is not affected by risk aversion, and (iii) in equilibrium, expected revenue is greater in FPSB than in SPSB*.

### 3.2 FPWP

We start by deriving a symmetric equilibrium for FPWP. If the other bidders stick to the same bidding function that is strictly increasing in type, a bidder with a type *v*, pretending to be a type $t\in [0,\bar{v}]$, faces the following problem:

$$\underset{t}{\max}\;U(v,t)=F^{\left(1\right)}(t)u(v-A(t)+\gamma t)+\int_{t}^{\bar{v}}u(\gamma \tilde{V}(x))dF^{\left(1\right)}(x)$$

where *F*^(1)^ denotes the distribution of the highest-order statistic of *n*−1 i.i.d. draws from *F*. The first term on the RHS refers to the case in which the bidder wins and then the outside observer induces that the bidder’s value equals *t*. The second term is the bidder’s payoff when losing the auction, where $\tilde{V}(x)$ denotes the outside observer’s optimal value estimate for losing bidders if the winning bidder’s value equals *x*. The equilibrium FOC is given by

$${\frac{\partial U(v,t)}{\partial t}|}_{t=v}=f^{\left(1\right)}(v)u(v-A(v)+\gamma v)-F^{\left(1\right)}(v)u\prime (v-A(v)+\gamma v)(A\prime (v)-\gamma )-u(\gamma \tilde{V}(v))f^{\left(1\right)}(v)=0$$

where *f*^(1)^ is the density function corresponding to *F*^(1)^.

The second-order condition for this equilibrium is satisfied as from *u*′>0, *u*′′<0, *f*^(1)^(*t*)>0, *F*^(1)^(*t*)>0, and *A*′(*t*)−*γ*>0 it follows that $sign(\frac{\partial U(v,t)}{\partial t})=sign(\frac{\partial U(v,t)}{\partial t}-{\frac{\partial U(t,v)}{\partial v}|}_{v=t})=f^{\left(1\right)}(t)\{u(v-A(t)+\gamma t)-u(t-A(t)+\gamma t)\}-F^{\left(1\right)}(t)(A\prime (t)-\gamma )\{u\prime (v-A(t)+\gamma t)-u\prime (t-A(t)+\gamma t)\}=sign(v-t)$.

The equilibrium uniquely follows from the differential equation with boundary condition *A*(0) = 0. It follows that

$$A^{\prime }(v)=\frac{f^{\left(1\right)}(v)}{F^{\left(1\right)}(v)}\frac{u(v(1+\gamma )-A(v))-u(\gamma \tilde{V}(v))}{u\prime (v(1+\gamma )-A(v))}+\gamma$$

$$>\frac{f^{\left(1\right)}(v)}{F^{\left(1\right)}(v)}(v(1+\gamma )-A(v)-\gamma \tilde{V}(v))+\gamma .$$ (1)

To establish (1), notice that $\frac{u(v)-u(z)}{u\prime (v)}>v-z$ for all *v*>*z* and that in equilibrium the winning payoff is always higher than the losing payoff. Then, using the same technical arguments as [23] (see Chapter 4, page 39), we get

A(v)>B(v)

for all *v*>0, where $B(v)=(\frac{n-1}{n}+\frac{n+1}{2n}\gamma )v$ represents the risk-neutral equilibrium of FPSP. We can then conclude with the following proposition:

#### Proposition 2

*If the winner identity and her payment are revealed to the outside observer, risk-averse bidders bid more aggressively than risk-neutral bidders in FPSB*.

Therefore, risk aversion may explain the overbidding observed for the FPWP in BGOT (see Table 1). As we will show in Section 3.4, the overbidding implied by risk aversion can be sufficiently strong to reverse the ranking of expected revenues between FPSB and SPSB, consistent with BGOT’s data.

### 3.3 SPWP

The analysis for SPWP proceeds analogously to that for FPWP. Let *V*(*x*) and *L*(*x*) denote the outside observer’s estimate of the winner’s value and the loser’s value respectively, conditional on the second-highest value being *x*. Recall that the identity of the second highest bidder is unknown to the outside observer. A type *v* loser is the second highest bidder with probability *H*(*v*) = (*n*−1)*F*^*n*−2^(*v*)(1−*F*(*v*)) and ranks third or lower with probability *K*(*v*) = (*n*−1)*F*^*n*−2^(*v*)−(*n*−2)*F*^*n*−1^(*v*). We let *h* and *k* denote the density functions associated with *H* and *K* respectively. Therefore, a bidder with a type *v*, pretending to be a type *t*, faces the following problem:

$$\underset{t}{\max}\;U(v,t)=\int_{0}^{t}u(v-A(x)+\gamma V(x))dF^{\left(1\right)}(x)+H(t)u(\gamma L(t))+\int_{t}^{\bar{v}}u(\gamma L(x))dK(x),$$

with $V(x)=\frac{\int_{x}^{\bar{v}}ydF(y)}{1-F(x)}$ and $L(t)=\frac{t}{n-1}+\frac{n-2}{n-1}\frac{\int_{0}^{t}xdF(x)}{F(t)}.$ The equilibrium FOC is given by

$${\frac{\partial U(v,t)}{\partial t}|}_{t=v}=f^{\left(1\right)}(v)u(v-A(v)+\gamma V(v))+h(v)u(\gamma L(v))+H(v)\gamma L^{\prime }(v)u^{\prime }(\gamma L(v))-k(v)u(\gamma L(v))=0,$$

from which the unique equilibrium bidding function *A* can be readily derived.

The second-order condition is satisfied because *u*′>0 and *f*^(1)^(*t*)>0 imply that $sign(\frac{\partial U(v,t)}{\partial t})=sign\left(\frac{\partial U(v,t)}{\partial t}-{\frac{\partial U(t,v)}{\partial v}|}_{v=t}\right)=f^{\left(1\right)}(t)\{u(v-A(t)+\gamma V(t))-u(t-A(t)+\gamma V(t))\}=sign(v-t)$.

In the remainder of our paper, we restrict attention to *F* = *U*[0, 1] and CARA preferences, i.e., *u*(*x*) = −exp(−*αx*)/*α*, *α*>0. *F* = *U*[0, 1] implies $V(v)=\frac{1+v}{2},L(v)=\frac{n}{2n-2}v,H(v)=(n-1)v^{n-2}(1-v)$, *K*(*v*) = (*n*−1)*v*^*n*−2^−(*n*−2)*v*^*n*−1^, and *F*^(1)^(*v*) = *v*^*n*−1^. The FOC becomes

$$\begin{array}{l} f^{\left(1\right)}(v)u(v-A(v)+\gamma V(v))+h(v)u(\gamma L(v))+H(v)\gamma L^{\prime }(v)u^{\prime }(\gamma L(v))-k(v)u(\gamma L(v))\\ \;=-(n-1)v^{n-2}\exp\left(\alpha A(v)-\alpha v-\alpha \gamma \frac{1+v}{2}\right)\\ \;-(n-1)v^{n-3}(n-2-(n-1)v)\exp\left(-\frac{\gamma \alpha n}{2n-2}v\right)\\ \;+(n-1)v^{n-2}(1-v)\frac{\gamma \alpha n}{2n-2}\exp\left(-\frac{\gamma \alpha n}{2n-2}v\right)\\ \;+(n-1)(n-2)v^{n-3}(1-v)\exp\left(-\frac{\gamma \alpha n}{2n-2}v\right)=0. \end{array}$$

Rearranging yields

$$A(v)=v-\frac{\gamma v}{2n-2}+\frac{\gamma }{2}+\frac{1}{\alpha }ln\{1+(1-v)\frac{\gamma \alpha n}{2n-2}\}.$$ (2)

Notice that *A*(*v*) is strictly decreasing in *α*>0. As the risk-neutral case is obtained in the limit for *α* = 0, it follows immediately that *A*(*v*)<*B*(*v*). This result is summed up in the following proposition:

#### Proposition 3

*Assume F* = *U*[0, 1] *and consider CARA preferences. If the winner identity and her payment are revealed to the outside observer, risk-averse bidders bid less aggressively than risk-neutral bidders in SPSB*.

Proposition 3 implies that for SPWP, equilibrium revenue for CARA risk preferences is lower than in the risk-neutral case. Intuitively, in SPWP, winning is relatively unattractive because the winner faces a random outside observer’s estimate as it depends on the second highest bid. Indeed, in the experimental data collected in BGOT, revenue in SPWP is much lower than in the risk-neutral equilibrium (see Table 1). This is a surprising result in view of the standard case without an outside observer in which bidding behaviour is not affected by risk aversion in SPSB.

### 3.4 Revenue comparison

We now compare FPSB and SPSB in terms of expected equilibrium revenue assuming *F* = *U*[0, 1] and CARA preferences. Let $R_{\alpha }^{T}$ denote the revenue for case *T*, *T* = FPW, FPWP, SPW, SPWP, where bidders’ degree of risk aversion equals *α*≥0, where *α* = 0 refers to the risk-neutral case. Let *A*_∞_ denote the bidding strategy for SPWP in the case of infinitely risk-averse bidders, i.e., $A_{\infty }(v)=\underset{\alpha \to +\infty }{\lim}A(v)$ for all *v*∈[0, 1]. Eq (2) implies

$$A_{\infty }(v)=v-\frac{\gamma v}{2n-2}+\frac{\gamma }{2}$$

because $\underset{\alpha \to +\infty }{lim}\frac{1}{\alpha }ln\left\{1+(1-v)\frac{\gamma \alpha n}{2n-2}\right\}=0$. It follows that the associated expected revenue, $R_{\infty }^{SPWP}$, is equal to $R_{\infty }^{SPWP}=\frac{n-1}{n+1}(1-\frac{\gamma }{2n-2})+\frac{\gamma }{2}$. Compared to the risk-neutral case, risk aversion affects equilibrium bidding strategies positively in FPSB and negatively in SPSB, and therefore can lead to a lower revenue in the latter:

$$R_{\infty }^{SPWP}=\frac{n-1}{n+1}+\frac{\gamma n}{2n+2}<R_{0}^{FPWP}=\frac{n-1}{n+1}+\frac{\gamma }{2}<R_{\alpha >0}^{FPWP}.$$

In other words, a sufficiently high level of risk aversion can explain the revenue ranking swap observed in BGOT’s data relative to the risk-neutral case. SPWP with infinitely risk-averse bidders also yields lower revenue than SPW:

$$R_{\infty }^{SPWP}=\frac{n-1}{n+1}+\frac{\gamma n}{2n+2}<R_{0}^{SPW}=R_{\alpha >0}^{SPW}=\frac{n-1}{n+1}+\gamma (k_{w}-k_{l}),$$

where the inequality follows from $k_{w}>\frac{n}{n+1}$ and $k_{l}<\frac{1}{2}\frac{n}{n+1}$.

Overall, while with risk-neutral bidders, $R_{0}^{SPW}<R_{0}^{SPWP}$ and $R_{0}^{FPWP}<R_{0}^{SPWP}$, for sufficiently risk-averse bidders, the opposite rankings are obtained:

#### Proposition 4

*Let F* = *U*[0, 1]. *For sufficiently risk-averse bidders with CARA preferences, SPWP yields lower revenue than SPW and FPWP, in contrast to the risk-neutral case*.

This result is qualitatively consistent with the data collected in BGOT (see Table 1). Therefore, risk aversion is again a potential candidate to explain the discrepancy between the data and the theoretical predictions. In SPW, equilibrium bids are unaffected: bidders still have a weakly dominant strategy to bid value plus the (deterministic) payoff from the outside observer providing a higher estimate for the winner than for the losers. As said, for SPWP, CARA bidders submit lower bids than risk-neutral bidders. We find that for sufficiently risk-averse bidders, SPWP yields lower expected revenue that SPW.

In contrast to SPWP, risk aversion produces an increase in equilibrium bids in FPWP compared to the risk-neutral case. The intuition is straightforward. First of all, like in the standard case, a bidder mitigates the risk of losing the auction by submitting a high bid. Second, in our setting, a risk-averse bidder has an additional incentive to win because the winner obtains a sure payoff from the outside observer’s estimate, which is deterministic for the winning bid, while a loser faces a stochastic estimate that depends on the winner’s bid. As a consequence, for sufficiently risk-averse bidders, the revenue rankings between SWP and SPWP on the one hand and between FPWP and SPWP on the other reverse, in line with BGOT’s experimental findings (see Table 1).

## 4. Conclusion

This paper has offered the equilibrium results for risk-averse bidders with signalling concerns, i.e., bidders who care about how they are perceived by an outside observer. Our results are twofold. First, unlike in the usual auction settings, when the winner’s identity and her payment are revealed, risk aversion yields less aggressive bidding behaviour in SPSB than in the risk-neutral case. Second, risk aversion is qualitatively consistent with several anomalies in [7]’s experiment. [7] find the ranking FPWP > FPW > SPW > SPWP while the ranking under risk-neutral bidders is SPWP > FPWP > FPW = SPW. In this paper, we have shown that risk aversion may explain the ranking reversal observed in the experiment.

Our study may inspire new research analysing the effect of risk aversion in other settings than the ones studied in this paper. New research could examine auction formats other than the FPSB and SPSB, like the all-pay auction or the English auction, as well as other information disclosure policies than revealing who wins the auction and how much the winner pays, including revealing the winning bid (rather than winner’s payment) or all bids. Such analysis would naturally fit the research agenda aimed at finding optimal auctions in settings where bidders have signalling concerns (see [5,8]).
